# Defect Structure Determination of GaN Films in GaN/AlN/Si Heterostructures by HR-TEM, XRD, and Slow Positrons Experiments

**DOI:** 10.3390/nano10020197

**Published:** 2020-01-23

**Authors:** Vladimir Lucian Ene, Doru Dinescu, Nikolay Djourelov, Iulia Zai, Bogdan Stefan Vasile, Andreea Bianca Serban, Victor Leca, Ecaterina Andronescu

**Affiliations:** 1 Department of Science and Engineering of Oxide Materials and Nanomaterials, Faculty of Applied Chemistry and Materials Science, University Politehnica of Bucharest, 060042 Bucharest, Romaniabogdan.vasile@upb.ro (B.S.V.); ecaterina.andronescu@upb.ro (E.A.); 2 Extreme Light Infrastructure-Nuclear Physics (ELI-NP), ‘Horia Hulubei’ National R&D Institute for Physics and Nuclear Engineering (IFIN-HH), 30 Reactorului Street, 077125 Măgurele, Romania; nikolay.djourelov@eli-np.ro (N.D.); iulia.zai@eli-np.ro (I.Z.); andreea.serban@eli-np.ro (A.B.S.); victor.leca@eli-np.ro (V.L.); 3 Doctoral School in Engineering and Applications of Lasers and Accelerators, University Politehnica of Bucharest, 060042 Bucharest, Romania; 4 Faculty of Physics, University of Bucharest, 077125 Măgurele, Romania

**Keywords:** gallium nitride, epitaxial thin films, dislocations, positron diffusion length

## Abstract

The present article evaluates, in qualitative and quantitative manners, the characteristics (i.e., thickness of layers, crystal structures, growth orientation, elemental diffusion depths, edge, and screw dislocation densities), within two GaN/AlN/Si heterostructures, that alter their efficiencies as positron moderators. The structure of the GaN film, AlN buffer layer, substrate, and their growth relationships were determined through high-resolution transmission electron microscopy (HR-TEM). Data resulting from high-resolution X-ray diffraction (HR-XRD) was mathematically modeled to extract dislocation densities and correlation lengths in the GaN film. Positron depth profiling was evaluated through an experimental Doppler broadening spectroscopy (DBS) study, in order to quantify the effective positron diffusion length. The differences in values for both edge $\left(\rho_{d}^{e}\right)$ and screw $\left(\rho_{d}^{s}\right)$ dislocation densities, and correlation lengths (*L*^e^, *L*^s^) found in the 690 nm GaN film, were associated with the better effective positron diffusion length (*L*_eff_) of $L_{\mathrm{eff}}^{\mathrm{GaN}2}$ = 43 ± 6 nm.

## 1. Introduction

Binary semiconductors, such as InN, AlN, GaAs, InAs, InP, GaN, AlSb, etc., and their alloys, cover an extended range of structures useful in high-end device technology [1,2]. Due to the direct bandgap that most of these materials possess, efficient emission and absorption of light is allowed. Many binary compounds also exhibit a very low electron effective mass, thus a high mobility, which makes them ideal candidates for developing high-speed devices [3]. Among these compounds, GaN has shown impressive advantages. Because of its geometric and electronic structure made up of covalent bonds between Ga and N, the wide energy band gap allows it to reach operating temperatures higher than 350 °C [2]. A second advantage is the high mobility (>1200 cm^2^ V^−1^ s^−1^) of the two-dimensional electron gas (formed at interfaces with e.g., AlN) that leads to low channel resistance and high current density (>1 A mm^−1^), and a breakdown field of 3.3 MV cm^−1^ that is 11 times higher than that of silicon (0.3 MV cm^−1^) [4,5]. GaN is widely used in applications that require either n-type or p-type doped semiconductors for charge carrier injection in different devices [6]. New methods of obtaining Ga based films using liquid Ga [7,8] for reactive depositions have emerged in recent years and the fundamentals behind liquid metal enabled synthesis, along with the related surface functionalization aspects [9] showed promising possibilities concerning the growth of GaN thin films. Despite this, the fabrication of defect-free GaN films still possesses interest in some fields, such as field assisted positron moderation [10].

Positron annihilation lifetime spectroscopy and Doppler broadening spectroscopy (DBS) have become the most used positron annihilation derived spectroscopy techniques suitable for non-destructive determinations of near surface crystallographic vacancies and dislocations in lattices, as well as optical and electronic properties of materials due to the high affinity of positrons to defects. Irrespective to the method used to obtain them, positrons manifest a broad energy distribution of about several hundreds of keV. In order to use the above-mentioned spectroscopy techniques for thin-film studies, positrons need to be moderated. The way to achieve this is to convert the fast positrons to slow positrons (with a low kinetic energy of few eV and a narrow bandwidth) by using a moderator material with negative work function for positrons (e.g., W or solid Ne) [11,12]. By varying the kinetic energy of the slow positrons, the depth at which they are implanted can be controlled [11]. The negative positron work function and the adequate branching ratio makes GaN a very promising candidate for field assisted positron moderation. A long positron diffusion length is expected due to the wide 3.4 eV bandgap. GaN studies have been undertaken and measurements have yielded values for the diffusion length of 19.3 ± 1.4 nm, surface branching ratio to free positrons of 0.48 ± 0.02 and positron work function of −2.4 ± 0.3 eV, respectively [13]. The moderator efficiency, usually smaller than 10^−2^, is greatly reduced by atomic scale defects which can trap positrons. 

GaN-based devices still encounter several obstructing issues, including high defect density and strain-induced polarization. In order to reduce the effects of these issues, a series of approaches were proposed in the last decade [1]. In the early stages, the main efforts were focused on improving both the qualities of the materials and the structuring of the device. The advanced growth techniques enabled management of the nanostructured layer interfaces, further enhancing the quantum efficiencies of devices. Substrates have a big influence on the growth mode and the final physical and chemical properties, determining the surface morphology, polarity, crystal orientation, composition, and elastic strains. When choosing a substrate, one of the most important criteria used is the mismatch parameter between the substrate and the deposited film. Lateral mismatch of lattices leads to a decrease of the thermal conductivity and accelerated diffusion of impurities. Vertical asymmetry causes a counter-phase interface. Thermal strain is induced in the film by the discrepancy between the thermal conductivities coefficients of substrates with respect to the epitaxial film. Chemical composition differences cause a contamination of the film which forms unstable electronic bonds and a mixed polarity that appears in the epitaxial film when the surface of the substrate is nonpolar [14]. Current reports of producing GaN films indicate that heteroepitaxial GaN films can be grown on different substrates such as Si, Al_2_O_3_, ZnO, TiO_2_, SiC, with different orientations [15]. The stable phase of gallium nitride is the α-phase wurtzite structure. However, epitaxial layers can be achieved with the coexistence of wurtzite and zinc-blende (β-phase) phases due to the stacking sequence of nitrogen and gallium atoms. Both structures have polar axes and they do not have an inversion symmetry [16]. 

The aim of this study is to assess the quality of commercially available GaN epitaxial thin films, grown on Si, for their potential use as positron moderators. High-resolution transmission electron microscopy (HR-TEM) and high-resolution X-ray diffraction (HR-XRD) were performed in order to determine the GaN films’ defect structures. The features of the heterostructures, such as layer thicknesses, interfaces, elemental diffusion, and dislocations were correlated with the effective positron diffusion lengths, evaluated by slow-positron DBS studies. 

## 2. Materials and Methods

### 2.1. Materials

Two gallium nitride, GaN, thin films grown using an epitaxial growth technique on Si substrates were used in this study. The wafers were acquired from NTT Advanced Technology Corporation (Kanagawa, Japan) and are defined by high uniformity, high breakdown voltage, a sheet carrier density of approximatively 10^13^ cm^−2^, and an electron mobility of over 2000 cm^2^ V^−1^ s^−1^. The two wafers, were further labeled as GaN300/Si and GaN700/Si, where the number stands for the claimed thickness of the GaN film, expressed in nm. No further details on structure, defects, and interfaces were made available by the producer. 

### 2.2. Structural Analysis 

#### 2.2.1. Microstructural Characterization

The microstructure of the wafers was studied with the help of a Titan Themis 200 image corrected transmission electron microscope (FEI, Hillsboro, OR, USA), equipped with a high-brightness field emission gun (X-FEG) electron source and a Super-X detector for energy dispersive spectroscopy (EDS). The heterostructures were investigated at 200 kV by HR-TEM, coupled with selected area electron diffraction (SAED) and scanning transmission electron microscopy (STEM) for elemental line profiling. Prior to analysis, the wafers were mechanically polished and then ion beam milled at a voltage of 3 kV and current of 5 mA until perforation. Ion-beam milling was continued with decrements of voltage and current, in order to remove debris produced by the high voltage ion beam thinning. 

For processing the elemental line profiles from EDS data, ImageJ software was used [17]. The visualization and analysis of crystal structures were made with SingleCrystal^®^ (Oxford, England), and images of simulated crystals were generated using CrystalMaker^®^, a software by CrystalMaker Software Ltd., Oxford, England [18].

#### 2.2.2. Defect Structure Determination

HR-XRD analysis was performed using a 9 kW Rigaku SmartLab diffractometer (Neu-Isenburg, Germany), with a rotating Cu anode (*K*_α_ = 1.5418 Å) and a HyPix-3000 high-resolution detector (Rigaku, Neu-Isenburg, Germany), in 0D mode. The data (*ω*—rocking curves of selected symmetrical and asymmetrical reflections) were recorded in double-axis configuration, in the parallel beam mode, using a parabolic mirror (cross beam optics module) and a four bounce Ge-220 monochromator (Rigaku, Neu-Isenburg, Germany), resulting in an axial divergence of the beam of 0.003° in the vertical diffraction plane of the goniometer. A narrow incidence slit of 1 mm was used to avoid the effect of sample curvature on the measurements. On the detector side, receiving slits (RS) of RS1 = 4 mm, and RS2 = 38.5 mm were used (open detector configuration), so that all diffuse scattering from the sample was accounted for. The wafers were first aligned with respect to the Si substrate, in order to avoid any measurement errors due to sample misalignment, then the rocking curve measurement of the selected GaN planes was performed. 

The recorded data was processed using the theoretical model developed by Kaganer et al. [19], using an integral of the form:

(1) $$I\left(\omega \right)=\frac{I_{i}}{\pi }\int_{0}^{\infty }\exp\left(-Ax^{2}\ln\left(\frac{B+x}{x}\right)\right)\cos\left(\omega x\right)dx+I_{\mathrm{backgr}}$$

where *I*_i_ is the integrated peak intensity and *I*_backgr_ is the background intensity. The *A* and *B* parameters were obtained by integral fitting on the experimental data. *A* and *B* describe the dislocation density and the dislocation correlation range, respectively, and can be expressed as:

(2) $$A=f\rho_{d}b^{2};\;B=\frac{gL}{b}$$

where *b* is the Burgers vector, $\rho_{d}$ is the dislocation density, *L* is the dislocation correlation length, *f* and *g* are two dimensionless parameters which depend on the skew geometry of the diffraction setup:

(3) $$f^{e}=\frac{0.7\cos^{2}\psi {\;\cos}^{2}\phi }{4\pi \cos^{2}\theta_{B}};\;f^{s}=\frac{0.5\sin^{2}\psi {\;\cos}^{2}\phi }{4\pi \cos^{2}\theta_{B}};\;g^{e}=\frac{2\pi \cos\theta_{B}}{\cos\phi \;\cos\psi },;\;g^{s}=\frac{2\pi \cos\theta_{B}}{\cos\phi \;\sin\psi }$$

where *ψ* is the angle between the sample surface and the scattering vector, *ϕ* is the angle between either incident or diffracted vector and the sample surface, and *θ*_B_ is the Bragg angle at which the diffraction interference takes place, according to the geometry described in Ref. [19]. Both *f* and *g* can be computed so that the density of dislocations, as well as the characteristic dislocation correlation length, can be obtained for either edge or screw defects, marked by the superscripts “e” and “s” in Equation (3). For edge dislocations, an asymmetrical lattice plane of the GaN network was considered, while for screw dislocations, a symmetrical plane of the same sample was used. For symmetric Bragg reflections (so, for screw dislocations), the setup implies that *ψ* = π/2 and *ϕ* = *θ*_B_, resulting in *f* = 1/8π and *g* = 2π, respectively [19].

### 2.3. Doppler Broadening Spectroscopy 

With a great probability, the annihilation of a positron with an electron in condensed matter is followed by the emission of two gamma rays of energy *E*_γ_ ≈ 511 keV. The longitudinal component of the annihilation pair momentum, *p*_L_, determines the energy shift due to Doppler broadening, Δ*E*_γ_ = 511-*E*_γ_ = *p*_L_*c*/2, where *c* is the speed of light. The Doppler broadening spectra of the annihilation radiation are sensitive to the electron momentum distribution of the site where the positron annihilated, since, the momentum distribution of the electrons in defects differs from that of electrons in the bulk material [20]. 

The DBS experiments were performed at the slow positron beam line of the Institute of High Energy Physics in Beijing, China. The gamma energy spectra were recorded by a HPGe detector (ORTEC, Zoetermeer, Netherlands), with a resolution of FWHM (full width at half maximum) = 0.97 keV estimated for 511 keV line. The detector was placed perpendicularly in respect to the positron beam axis, at a distance of 20 cm from the sample. The incident positron energy was controlled from *E*_+_ = 0.5 to 25 keV. Each of the experimental spectra was collected over a period of 8 min for a fixed *E*_+_, resulting in statistics of ~5 × 10^5^ counts in the 511 keV region. The shape of the annihilation peak was analyzed by the sharpness parameter, *S*, defined as the sum of counts, in the central region of the peak (|Δ*E*_γ_| < 0.78 keV), relative to the total peak counts (*N*_tot_), determined in the range between 500 and 522 keV. The triplet state of positronium (Ps) decays by emitting 3-gamma rays when it does not interact with the electrons of the material. The ratio, *F*_Ps_, between the counts in the valley region (from 450 to 500 keV) in the energy spectrum to *N*_tot_ can give a relative estimate of the Ps emitted from the surface. 

The implantation profile of positrons in a material with density ρ in g cm^-3^ can be described, according to Ref. [21], by:

(4) $$P\left(z,E_{+}\right)=\frac{2z}{z_{0}}\exp\left(-{\left(\frac{z}{z_{0}}\right)}^{2}\right)$$

where *z* is the depth at which the positron is located, expressed in nm, *z*_0_ *=* 1.13 *z*_m_, and the mean penetration depth is

(5) $$z_{m}=\;(36/\rho )E_{+}^{1.62}\;\mathrm{nm}$$

Different layer densities are taken into account by using the modified positron implantation profile described by:

(6) $$P_{\rho }\left(z_{\rho },E_{+}\right)=\rho \left(z_{\rho }\right)/\rho_{0}P\left(z,E_{+}\right)$$

with $=\int_{0}^{z_{\rho }}\rho \left(\zeta \right)/\rho_{0}d\zeta$, where *ρ*_0_ is the density of the substrate. In the analysis of the experimental data, densities of 2.33, 3.26 and 6.15 g cm^−3^ were used for the Si substrate, AlN buffer layer, and GaN film, correspondingly. 

Due to the correlation between the mean positron implantation depth, *z*_m_, and *E*_+_, the experimental data *S*(*E*_+_) and *F*_Ps_(*E*_+_) represents depth profiles. The VEPFITsoftware (Delft University of Technology, Delft, Netherlands) was used to fit the experimental data [22]. In addition to the implantation, the processes that have to be taken into account to solve the positron transport problem are diffusion, drift (in case of electric field), and trapping or annihilation of free positrons. Surface related processes, such as Ps emission and positron surface trapping, are incorporated within the model. The influence of epithermal positrons, and that of thermal positrons which diffuse back to the surface, is also taken into account in the VEPFIT software. 

The *S*(*E*_+_) is fitted using a model described by:

*S*(*E*_+_) = *S*_e_*F*_e_(*E*_+_) + *S*_s_*F*_s_(*E*_+_) + ∑ *S*_i_*F*_i_(*E*_+_) (7)

with *F*_e_(*E*_+_) + *F*_s_(*E*_+_) +∑ *F*_i_(*E*_+_) = 1, where *F*_e_(*E*_+_) is the fraction of epithermal positrons annihilated at the surface, and *F*_s_(*E*_+_) and *F*_i_(*E*_+_) are the fractions of thermalized positrons annihilated at the surface and in the i-th layer. *S*_e_, *S*_s_, and *S*_i_ are characteristic parameters, respectively, corresponding to the annihilation of epithermal positrons and of thermalized positrons at the surface and in the bulk of i-th virtually uniform layer. VEPFIT uses discretization as a fast method of solving numerically the positron transport problem to obtain the fractions of annihilated positrons from the above described states. One of the parameters which is derived from the fit is the effective positron diffusion length (*L*_eff_) for each layer. *L*_eff_ is limited by the layer defects and is described by:

(8) $$L_{\mathrm{eff}}=\left[D^{+}/\left(k_{t}n_{t}+\lambda_{b}\right)\right]$$

where *D*^+^ is the positron diffusion coefficient, *λ*_b_ is the annihilation rate of positrons in a defect-free material, and the product between the defect density, *n*_t_, and the positron trapping rate, *k*_t_, for vacancies, usually holds the value of 10^15^ s^−1^.

Often, the information of the Ps emission from the surface, as derived from *F*_Ps_(*E*_+_), is useful in the interpretation of the experimental results. Both depth profiles *S*(*E*_+_) and *F*_Ps_(*E*_+_) can be fitted simultaneously by one and the same VEPFIT model. 

## 3. Results and Discussion

### 3.1. Microstructural Characterization 

#### 3.1.1. TEM

Upon analyzing the structure of the two wafers, the existence of an AlN buffer layer was acknowledged. Such a buffer layer has the purpose of accommodating the GaN network to that of the Si substrate, thus decreasing the film strain and the amount of defects that would be generated during film growth due to lattice mismatch [23]. Although the lattice mismatch between GaN and Si ($11\bar{2}1$) is lower (16.9%) than in the case of AlN and Si ($11\bar{2}1$) (18.9%), the use of an AlN buffer layer is still recommended due to the low mismatch between AlN and GaN (2.4%) that can ultimately lead to a lower amount of defects in the final GaN film [24].

**Figure 1:**
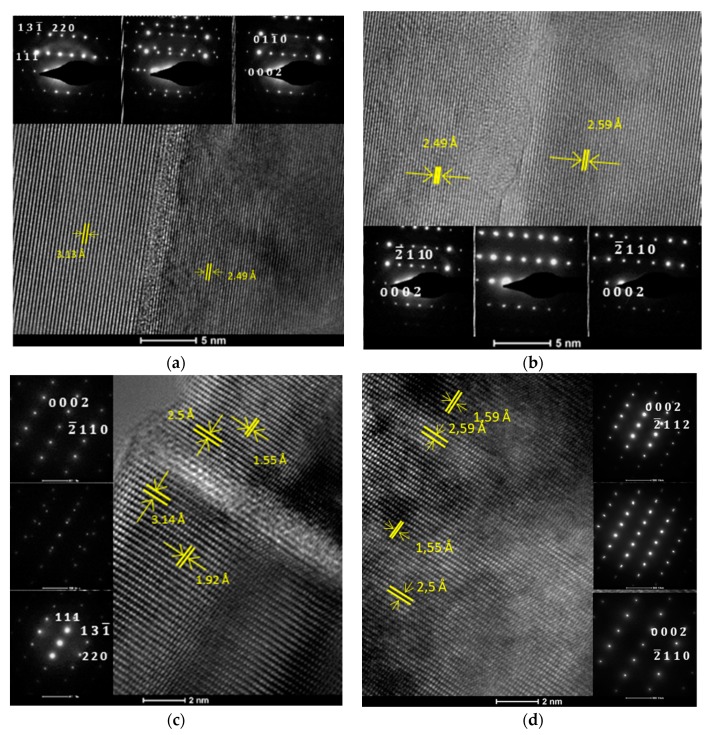
High-resolution transmission electron microscopy (HR-TEM) micrographs and selected area electron diffraction (SAED) patterns showing the display of atom planes in respect to their respective interfaces for: (**a**) GaN300/Si–Si/AlN interface, (**b**) GaN300/Si–AlN/GaN interface, (**c**) GaN700/Si–Si/AlN interface, (**d**) GaN700/Si–AlN/GaN interface.

The TEM and SAED images in Figure 1 show the display of planes near the Si/AlN interface and near the AlN/GaN interface in the GaN300/Si and GaN700/Si samples.

Regarding the substrates from both wafers, the interplanary distance of 3.13 Å, corresponding to ($11\bar{2}1$) planes, confirm the $Fd\bar{3}m$ diamond-like cubic structure of Si (International Centre for Diffraction Data [ICDD] 00-005-0565), whereas the interplanary distance of 2.49 Å, corresponding to (0 0 0 2) planes, confirmed the *P63mc* hexagonal structure of the AlN (ICDD 00-025-1133) buffer layer. Literature studies revealed that GaN has a better affinity to grow on Si ($11\bar{2}1$) rather than Si (0 0 0 1) because of the threefold symmetry of the Si ($11\bar{2}1$) and the six-fold arrangement for Si atoms that are present in the case of growing AlN/GaN along the (0 0 0 2) direction [25]. From the SAED patterns in Figure 1a,c, it can be deduced that through a semi-coherent interface of about 1 nm, containing point defects and a low degree of crystallinity, hexagonal AlN grew over cubic Si, with the relationship $Fd\bar{3}m$ Si ($11\bar{2}1$) || (0 0 0 2) AlN *P63mc*. Interplanary distances of 2.49 Å, corresponding to (0 0 0 2) planes, highlight once more the *P63mc* hexagonal structure of AlN. Regarding the film, interplanary distances of 2.59 Å, corresponding to (0 0 0 2) planes, confirm the *P63mc* hexagonal structure of GaN (ICDD 00-050-0792). From the SAED patterns in Figure 1b,d, it can be deduced that through an interface containing linear dislocations, hexagonal GaN grew over hexagonal AlN with the relationship *P63mc* AlN (0 0 0 2) || (0 0 0 2) GaN *P63mc*. In comparison with the thinner GaN film (GaN300/Si), the thicker one (GaN700/Si), although it possesses the same crystallographic relationship relative to the buffer layer, shows fewer point defects and linear dislocations at the interface, most likely due to the higher amount of time needed to deposit a thicker film, during which, the sample is kept, in the manufacturing process, at a temperature that favors the dislocation movement under thermal stress [26].

**Figure 2:**
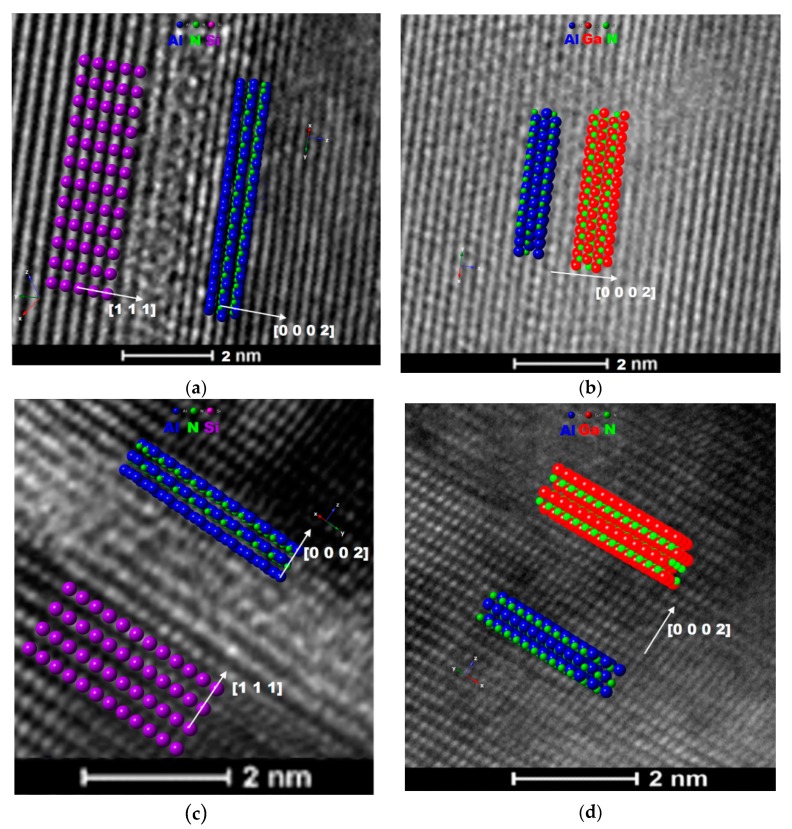
HR-TEM micrographs with display of simulated crystal lattices near the interface between Si substrate and AlN buffer layer in (**a**) GaN300/Si, (**c**) GaN700/Si and between AlN buffer layer and GaN film for (**b**) GaN300/Si, (**d**) GaN700/Si.

Simulated crystal models, based on the SAED patterns are presented in Figure 2, as overlays on the HR-TEM micrographs of the interfaces.

**Figure 3:**
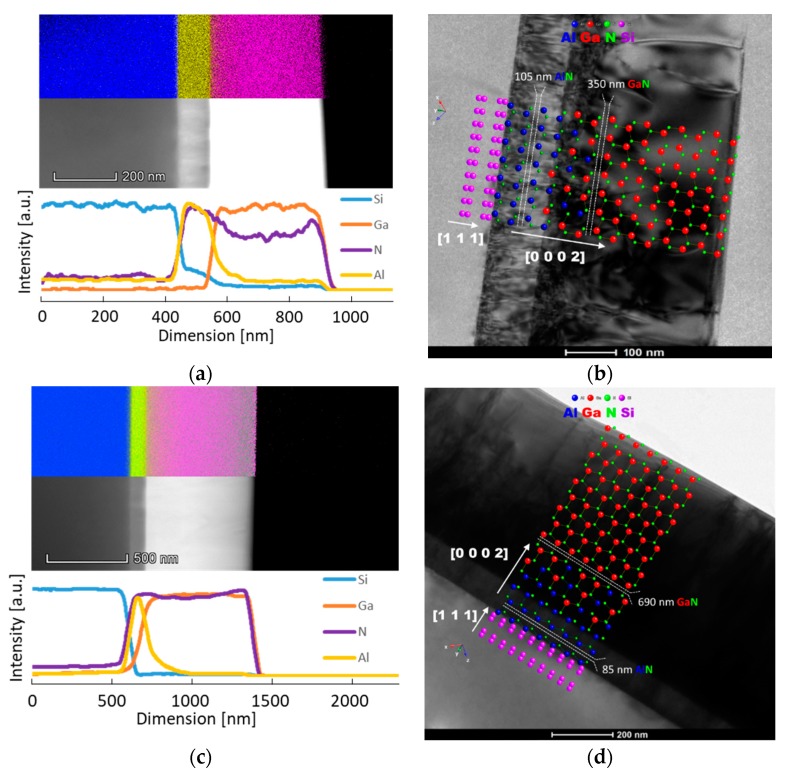
STEM micrographs with EDS mapping and elemental line profiles for (**a**) GaN300/Si, (**c**) GaN700/Si, and TEM micrographs showing the overview of the two wafers, (**b**) and (**d**), respectively.

In order to assess layer thicknesses and elemental diffusion length, TEM, STEM and EDS were performed, which are highlighted in Figure 3.

The STEM study allowed assessing layer thicknesses for both samples, the thinner one having a 350 nm GaN film and a 105 nm AlN buffer layer, whereas for the thicker sample, a 690 nm GaN film and an 85 nm AlN buffer layer were found. For both samples, the epitaxial growth relationship can be described by the relationship: GaN P63mc (0 0 0 2) || P63mc AlN (0 0 0 2) || ($11\bar{2}1$) $Fd\bar{3}m$ Si.

From the EDS maps and elemental line profiles presented in Figure 3a,c, it can be seen that both samples manifested Al diffusion at the edge of the GaN layer. Because the film remained highly crystalline and the interface between GaN/AlN is highly coherent, the diffusion of Al is most likely due to the native defects mediated by Al displacements either during high-temperature annealing of the film [27] or a film growth process that involves high temperatures. 

#### 3.1.2. XRD

**Figure 4:**
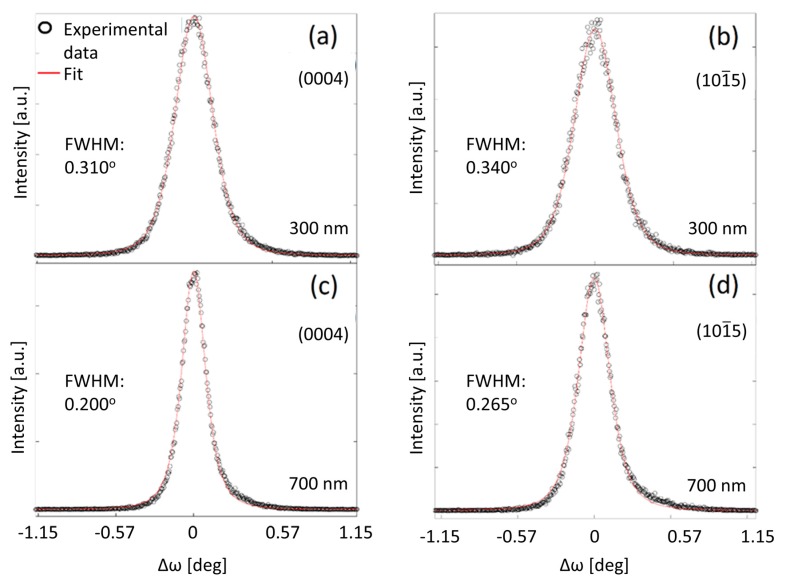
Experimental and simulated omega scans around (**a**) (0 0 0 4) planes of GaN in GaN300/Si, (**b**) ($10\bar{1}5$) planes of GaN in GaN300/Si, (**c**) (0 0 0 4) planes of GaN in GaN700/Si, (**d**) ($10\bar{1}5$) planes of GaN in GaN700/Si. Abbreviations: FWHM, full width at half maximum.

In order to assess the threading dislocation density and correlation length of the GaN films, two pairs of rocking curves (*ω* scans) were measured: one for the (0 0 0 4) plane, to assess the screw values $\rho_{d}^{s}$ and *L*^s^, and another one for the ($10\bar{1}5$) plane, to determine the edge characteristics $\rho_{d}^{e}$ and *L*^e^. The collected and simulated omega scans, along with their respective full width at half maximum (FWHM), are shown in Figure 4. 

The experimental advantage of the open detector consists in obtaining high intensities, while the mathematical interpretation implies a simpler intensity distribution that can be described using a one-dimensional integral. While the model usually applies for any diffraction maximum, separation between two peaks that are in close proximity of each other requires a triple-axis configuration of the diffractometer, with analyzer at the detector side, making the mathematical processing in this case more complex, requiring a fit of a two-dimensional integral [19]. The (0 0 0 4) and ($10\bar{1}5$) planes are chosen because of lack of overlapping diffraction peaks near the respective *ω* (coming from either the buffer layer or substrate).

The length of the Burgers vector of edge dislocations was *b*^e^ = 0.32 nm and for screw dislocations - *b*^s^ = 0.52 nm. Parameters *f*^e^ and *g*^e^, *f*^s^, and *g*^s^ are calculated with Equation (3), and with the help of the extracted *A* and *B*, a series of threading dislocation densities and correlation lengths were calculated, the results being summarized in Table 1. The total threading dislocation density, $\rho_{d}^{t}$, is calculated as the sum of the two component densities (screw and edge), while the mean distance between two dislocations is given by *r*_d_ = 1/${\left(\rho_{d}^{t}\right)}^{1/2}$ [28]. As shown in Table 1, the thicker GaN film manifests defect densities $\rho_{d}^{e}$ = 2.24 × 10^11^ cm^−2^ and $\rho_{d}^{s}$ = 1.35 × 10^10^ cm^−2^, both lower than those of the thinner one, $\rho_{d}^{e}$ = 4.19 × 10^11^ cm^−2^ and $\rho_{d}^{s}$ = 1.85 × 10^10^ cm^−2^. In the GaN700/Si wafer, the values for the dislocations correlation lengths, *L*^e^ = 41 nm and *L*^s^ = 220 nm, are higher compared to the corresponding values of the GaN300/Si wafer, *L*^e^ = 27 nm and *L*^s^ = 107 nm. The dislocation correlation length, also known as screening range, corresponds to the average size of cells in which the total Burger vector is equal to zero. The correlation lengths values suggest a reduced scattering of X-rays for the GaN film from the GaN700/Si wafer, also indicated by the smaller values of the FWHM, depicting a better quality of the film.

### 3.2. Positron Implantation Profile

**Figure 5:**
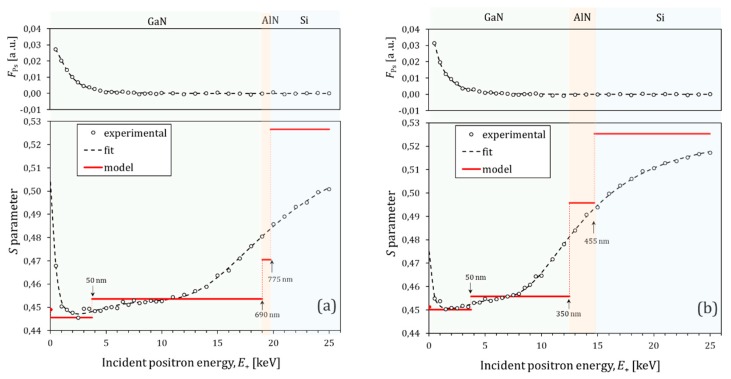
Plotted depth profiles *S*(*E*_+_) of (**a**) GaN700/Si and (**b**) GaN300/Si. The experimental errors are in the order of the experimental point size. The stairs represent the best parameters obtained by the fit of a 4-layer model to the experimental data by the VEPFIT software. The upper part of figure is the experimental data and the best fit of the relative Ps fraction, *F*_Ps_(*E*_+_).

The depth profiles *S*(*E_+_*) for the GaN300/Si and GaN700/Si are shown in Figure 5. The sharp initial decrease of *S* for *E*_+_ ≲ 1 keV was due to annihilated epithermal positrons. Because of their high kinetic energy in the moment of annihilation, *S*_e_ did not reflect the material structure. At *E*_+_ ≳ 1 keV, it can be seen that *S* slowly increased with *E*_+_ in the GaN film range, while approaching the AlN buffer layer, a stronger increase starts (at *E*_+_ ≳ 11 keV) and tends to reach a saturation level in the Si substrate (better seen in Figure 5b). Full saturation can be expected at high enough energies (*E*_+_ > 25 keV) to have all implanted positrons annihilated entirely in the Si substrate. 

Based on the TEM information for the wafer layers, a three-layer (GaN, AlN, and Si) model was applied to fit the experimental *S* parameter by VEPFIT. The effective positron diffusion length in the Si substrates was fixed to 245 nm in accordance to available literature data [29,30]. The thicknesses of layers in the model were fixed to the values determined by the TEM analysis (see Section 3.1.1). The boundary depths of the layers were calculated by Equation (5) and indicated in Figure 5. These preliminary fits, for both samples, resulted in normalized chi squares (*χ*^2^) of 1.47 and 1.40 for GaN700/Si and GaN300/Si, respectively, and also, into long *L*_eff_ ~ 100 nm for the GaN film in both cases. The curves of the preliminary fits were very close to the fits showed in Figure 5. However, the best fit parameters revealed that the *S*_s_ ~ 0.445 was found to be lower than *S*_GaN_ ~ 0.455 (specific to positrons annihilated in GaN film). No Ps was formed in the bulk of GaN, however, at the surface, the branching ratio showed that 12% of the positrons formed Ps [13]. The triplet state of Ps (*o*-Ps) annihilates in vacuum into three gamma rays that do not contribute to the 511-keV peak. Statistically, 25% of Ps is singlet form (*p*-Ps). The *p*-Ps annihilation in vacuum was characterized by a narrow Doppler shift distribution curve, thus, with a high *S* [31]. For sample-detector longitudinal geometry, the emission of Ps at low incident positron energy may cause asymmetry in the Doppler broadened peak [32]. It is caused by a shift of the centroid of the *p*-Ps contribution and may lead to an increase of the annihilation peak width. However, for the geometry described in Section 2.3, if Ps is emitted from the surface, the centroid of the *p*-Ps contribution will not be shifted. Therefore, *S*_s_ should have a larger value than *S*_GaN_. As this relationship is not fulfilled for the preliminary fit results, it can be concluded that this type of fit is physically incorrect. Our attempts to force *S*_s_ to be greater (or at least equal) than *S*_GaN_, without any increase in the number of layers of the model, led to bad fits with *χ*^2^ > 7. The latter indicates depth inhomogeneity of the GaN film. 

In a defect-free GaN, produced by metalorganic vapor phase epitaxy, *S* decreased smoothly with the increase of *E*_+_, which is typical for materials with long effective positron diffusion length (reported as $L_{\mathrm{eff}}^{\mathrm{DF}}$ = 135 nm) [33]. For Mg-doped p-type GaN, a sharp decrease in *S* was observed for *E*_+_ ≲ 1 keV, similarly to what can be seen in Figure 5. Uedono et al. explain this behavior by the created local electric field due to band bending near the surface, which suppresses the back diffusion of the thermalized positrons to the surface [34]. As a result, less Ps is formed at the surface by the thermalized positrons, and the positron diffusion length is shortened in a near surface layer. Based on the above reasoning, the number of the layers of the fitting model is changed by splitting the GaN film into sublayers (GaN1 and GaN2). In order to have a better understanding of the near surface positron annihilation, simultaneous fit of *S*(*E*_+_) and *F*_Ps_(*E*_+_) were performed by VEPFIT. Reasonable fits (see Figure 5) were obtained with two sublayers of the GaN film (4-layer model). The best fit parameters are summarized in Table 2. 

In a material in which Ps is not formed in the bulk, a higher *S* parameter means either more defects or bigger defects [34]. Also, more defects or more efficient positron trapping by defects (occurs for bigger defects) will result, according to Equation (4), in shorter *L*_eff_. For GaN700/Si, the value *S*_GaN1_ = 0.4456 ± 0.0004 is lower than *S*_GaN2_ = 0.4456 ± 0.0004 and this relationship indicates lower quality of the GaN2 sublayer compared to GaN1. The fact that $L_{\mathrm{eff}}^{\mathrm{GaN}1}$ = 13.1 ± 0.4 nm is shorter than $L_{\mathrm{eff}}^{\mathrm{GaN}2}$ = 43 ± 6 nm seems to contradict the latter statement. However, the short $L_{\mathrm{eff}}^{\mathrm{GaN}1}$ can be explained by the presence of local electric field directed inward the surface. Using the detector resolution and the *S* determination range given in Section 2.3, the characteristic parameter for *p*-Ps annihilation, *S_p_*_-Ps_, was estimated to be 0.95. If the branching ratio of Ps formation by thermalized positrons on the surface is 12% [13], the *p*-Ps annihilation contribution will be 3%. This should lead to an 0.028 increase in *S*_s_, compared to *S*_GaN1_. As can be seen in Figure 5b, the *S*_s_ (see the parameter’s stair at *E*_+_ = 0) was very close to *S*_GaN1_, indicating strong reduction in the Ps formation due to back-diffusion of thermalized positrons to the surface. The results for GaN300/Si in Figure 5a can be explained analogously.

For both samples, $L_{\mathrm{eff}}^{\mathrm{GaN}2}$ (see Table 2) are shorter than the defect free value $L_{\mathrm{eff}}^{\mathrm{DF}}$ = 135 nm. Saleh and Elhasi [35] suggested that the observed low *L*_eff_ < 60 nm values of positron diffusion length in GaN are due to positron interaction with dislocations. The dislocations can shorten *L*_eff_ by enhanced scattering of thermal positrons on them, and while vacancies tend to reside along them, they induce negative charge densities [36], trapping positrons more efficiently. In the case of the present study, the TEM analysis and the XRD defect assessment pointed out higher dislocation densities in the GaN film of the GaN300/Si wafer compared to GaN700/Si wafer (see Table 1). This is in agreement with the shorter effective positron diffusion length $L_{\mathrm{eff}}^{\mathrm{GaN}2}$ = 22 ± 6 nm (higher $S_{\mathrm{GaN}2}$ = 0.4558 ± 0.0004) in the GaN300/Si wafer, compared to $L_{\mathrm{eff}}^{\mathrm{GaN}2}$ = 43 ± 6 nm ($S_{\mathrm{GaN}2}$ = 0.4536 ± 0.0003) in GaN700/Si wafer. Another explanation for the last relationships could be that the highly defect GaN/AlN interface region in the GaN film, has stronger influence on the *S*_GaN2_ and $L_{\mathrm{eff}}^{\mathrm{GaN}2}$ for the thinner GaN film.

It is important to note that the above VEPFIT analysis, summarized in Table 2, was done without considering any electric field or interface layers (interpenetrating) neither between GaN and AlN nor between AlN and Si. The presence of such interpenetrating interface layers were derived from the elemental line profiles in Figure 3 (better seen in Figure 3a). The significant polarization, due to charge densities present at semiconductor heterojunction interfaces, creates an internal electric field which influences the positron transport trough the heterojunction interface. The mechanism of forming the potential barrier is due to the equalization of the Fermi levels of the two materials by charge transfer [37]. The positrons cannot diffuse equally well in both directions across such interface. For example, the diffusing positrons in the GaN layer are pushed back by the potential barrier at the interface, while these which diffuse in AlN will fall in a well at the barrier. The positrons tend to localize at the well barrier interface in a nitride heterostructure, as shown by theoretical calculations [38], and observed experimentally for GaN/SiC hetrojunction [39]. These effects can lead to a wrong estimation by VEPFIT analysis of both thicknesses of the layers and effective positron diffusion lengths. However, in our case, the *S*(*E*_+_) points increased rather smoothly with the increase of *E*_+_ in the region of the AlN buffer layer (see Figure 5a,b) and the specific parameters for the AlN (see Table 2) are determined by large uncertainties even with no electric field. So, further complications of the model are not reasonable to be applied. 

## 4. Conclusions

Two commercially available GaN/AlN/Si wafers were characterized by means of TEM and XRD in order to assess the relationship of the heterostructures characteristics (i.e., thickness of layers, crystal structures, preferred orientation growth, elemental diffusion, edge, and screw dislocation densities) with the positron diffusion depths, evaluated by DBS studies. Although epitaxial films show, in general, a high periodicity in the crystal lattice, there are inevitable defects that are bound to appear due to lattice mismatch between substrates, buffer layers, and films. Hence, within the epitaxial layers, defined by a [GaN P63mc (0 0 0 2) || P63mc AlN (0 0 0 2) || ($11\bar{2}1$) $Fd\bar{3}m$ Si] relationship, a correlation between elemental diffusion, dislocation densities, and positron depth profiles was assessed. XRD dislocation evaluations pointed out higher density of dislocations in the GaN300/Si wafer ($\rho_{d}^{e}$ = 4.19 × 10^11^ cm^−2^, $\rho_{d}^{s}$ = 1.85 × 10^10^ cm^−2^, $\rho_{d}^{t}\;$= 4.37 × 10^11^ cm^−2^), implying a lower quality of the GaN film, compared to the one in the GaN700/Si wafer ($\rho_{d}^{e}$ = 2.24 × 10^11^ cm^−^^2^_,_ $\rho_{d}^{s}$ = 1.35 × 10^10^ cm^−^^2^, $\rho_{d}^{t}$ = 2.35 × 10^11^ cm^−^^2^). This was also supported by the higher dislocation correlation lengths found in the GaN700/Si wafer (*L*^e^ = 41 nm and *L*^s^ = 220 nm) as well as the larger mean distance between two dislocations (*r*_d_ = 21 nm) which corresponded to larger average size of cells in which the total Burger vector is equal to zero, implying a higher crystallinity of the GaN film, compared to the one in the GaN300/Si (*L*^e^ = 27 nm, *L*^s^ = 107 nm, *r*_d_ = 15 nm). Elemental diffusion studies carried out by TEM have shown that outside each layer boundary, both Al and Ga cross their respective layer interface to a certain depth, justifying the need of using a model that includes two different GaN layers (for each wafer) to explain the results from the DBS studies. Because both wafers were grown using the same method, in similar conditions, the improvement in crystallinity of the top GaN film is associated with the decreased lengths for elemental interfusion, relative to the GaN film width. While atomic displacements intermediate defect formation and propagation, a shorter length of non-stoichiometry in the GaN film induces a better quality of the top film, lowering the amount of defects and thus improving the positron moderation capacity of the material. The studied materials, because of their high amounts of edge and screw dislocations, diffusion, and partial non-stoichiometry, still imply several limitations in their use in the field of positron moderation. The positron data revealed the lack of uniformity in defect depth distribution, a fact that could not be observed in HR-TEM, nor in XRD. Therefore, using a positron-based complementary technique holds significant value for structural characterization. The DBS experiment assessed the effective positron diffusion length for both wafers, with a larger value of $L_{\mathrm{eff}}^{\mathrm{GaN}2}$ = 43 ± 6 nm ($S_{\mathrm{GaN}2}$ = 0.4536 ± 0.0003), corresponding to the GaN film found in the GaN700/Si wafer, compared with $L_{\mathrm{eff}}^{\mathrm{GaN}2}$ = 22 ± 6 nm ($S_{\mathrm{GaN}2}$ = 0.4558 ± 0.0004) for the GaN film in the GaN300/Si wafer.

## Figures and Tables

**Table 1:** Dislocation densities and correlation lengths for GaN in the GaN300/Si and GaN700/Si samples. The uncertainty of the presented values is within the least significant digit.

Sample	$\rho_{d}^{e}$ [cm^−2^]	$\rho_{d}^{s}$ [cm^−2^]	$\rho_{d}^{t}$ [cm^−2^]	*r*_d_ [nm]	*L*^e^ [nm]	*L*^s^ [nm]
GaN300/Si	4.19 × 10^11^	1.85 × 10^10^	4.37 × 10^11^	15	27	107
GaN700/Si	2.24 × 10^11^	1.35 × 10^10^	2.35 × 10^11^	21	41	220

**Table 2:** Best fit parameters obtained by VEPFIT from the *S*(*E*_+_) and *F*_Ps_(*E*_+_) depth profiles. The values without error margins are fixed parameters.

Sample		GaN300/Si *χ*^2^ = 1.15			GaN700/Si *χ*^2^ = 1.73		
Layer		*L*_eff_ [nm]	*S*	*d* [nm]	*L*_eff_ [nm]	*S*	*d* [nm]
GaN	Sublayer						
	GaN1	14.3 ± 0.5	0.4501 ± 0.0006	50	13.1 ± 0.4	0.4456 ± 0.0004	50
	GaN2	22 ± 6	0.4558 ± 0.0004	300	43 ± 6	0.4536 ± 0.0003	640
AlN		26 ± 10	0.4957 ± 0.0019	105	4 ± 33	0.4707 ± 0.0032	85
Si		245	0.5254 ± 0.0005	-	245	0.5264 ± 0.0011	-
